# Role of the environment in transmission of Gram-negative bacteria in two consecutive outbreaks in a haematology-oncology department

**DOI:** 10.1016/j.infpip.2022.100209

**Published:** 2022-02-25

**Authors:** W.C. van der Zwet, I.E.J. Nijsen, C. Jamin, L.B. van Alphen, C.J.H. von Wintersdorff, A.M.P. Demandt, P.H.M. Savelkoul

**Affiliations:** a Dept. Medical Microbiology, Maastricht University Medical Center, Maastricht, the Netherlands; b Dept. Haematology, Maastricht University Medical Center, Maastricht, the Netherlands

**Keywords:** Water points, Gram-negative bacteria, Nosocomial infection

## Abstract

In 2019–2020, two subsequent outbreaks caused by phenotypically identical ESBL-producing *Enterobacter cloacae* and multi-drug-resistant (MDR) *Pseudomonas putida* were detected in respectively 15 and 9 patients of the haematology-oncology department. Both bacterial species were resistant to piperacillin-tazobactam, used empirically in (neutropenic) sepsis in our hospital, and ciprofloxacin, used prophylactically in selective digestive decontamination for haematology patients. The *E. cloacae outbreak* was identified in clinical cultures of blood and urine. Despite intensified infection control measures, new cases were found in weekly point-prevalence screening cultures. Environmental samples of sinks and shower drains appeared positive in 18.1%. To diminish the environmental contamination burden, all siphons of sinks were replaced, and disinfection of sinks and shower drains was intensified using chlorine and soda on a daily basis. Replacement of shower drains was not possible. The outbreak of *P. putida* remained limited to rectal cultures only, and disappeared spontaneously without interventions. During both outbreaks, multiple strains of the incriminated bacterium were found simultaneously (demonstrated by Amplified-Fragment Length Polymorphism and/or Whole-Genome Multi-locus Sequencing Typing) in patients as well as the environment. It was experimentally shown that a biofilm on the toilet edge may act as a source for nosocomial transmission of Gram-negative bacteria. In conclusion, the drainage system of the hospital is an important reservoir of MDR bacteria, threatening the admitted patients. In existing hospitals, biofilms in the drainage systems cannot be removed. Therefore, it is important that in (re)building plans for hospitals a plan for prevention of nosocomial transmission from environment to patients is incorporated.

## Introduction

In recent years, many studies have demonstrated that sink drains, shower drains and toilets in hospitals can serve as a reservoir of Gram-negative bacterial infections in patients [[1], [2], [3]]. These bacteria reside in biofilms in and on water related devices, which are refractory to disinfection. Bacteria in the biofilm of drainage systems are under a prolonged antibiotic selective pressure, because antibiotics in faeces and urine of patients are absorbed by the biofilm [4]. Within these biofilms, containing several bacterial species, acquired mobile resistance elements are frequently transferred, resulting in highly resistant microorganisms, such as extended-spectrum β-lactamase and carbapenemase-producing Enterobacterales and Pseudomonas species [5,6].

Highly resistant, (as well as antibiotic susceptible), bacteria from biofilms can be transmitted to patients directly or indirectly via droplets, and go on to cause infections [7]. Furthermore, there have been a few reports of transmission via aerosols, but these remain a minority [8]. This transmission results in a serious threat to patients, especially critically-ill patients and immunocompromised hosts, leading to substantial morbidity and mortality, because treatment options are limited. Most Gram-negative healthcare-associated waterborne infections go undetected, unless they occur in outbreaks [2].

In Gram-negative nosocomial outbreaks, isolates found in patients and water-points can be identical, but the direction of transmission is usually unclear (water-point contamination originates from waste-water from the patient, or opposite direction). Therefore, the microbiomes of patients and water-points seem to be a continuum [9].

Usually, biofilms contain several bacterial species. The complete removal of biofilms from drains is often not possible, because they extend into the deeper parts of the water system, which cannot be reached. From this deeper compartment, biofilms grow back to the outlets, even from different patient rooms. Therefore, for infection control, mechanical cleaning and disinfection of the outlets of the system is often the maximum achievable goal.

We describe two subsequent outbreaks in 2019–2020 caused by ESBL-producing *Enterobacter cloacae* and multi drug-resistant *Pseudomonas putida* in the haematology-oncology ward of our hospital, which were related to contaminated water points. Both bacterial species were resistant to the antibiotics used for selective digestive decontamination (SDD), which is used to prevent systemic Gram-negative infection in neutropenic haematology patients, and also to the empirical antibiotic therapy for neutropenic fever.

**Figure 1:**
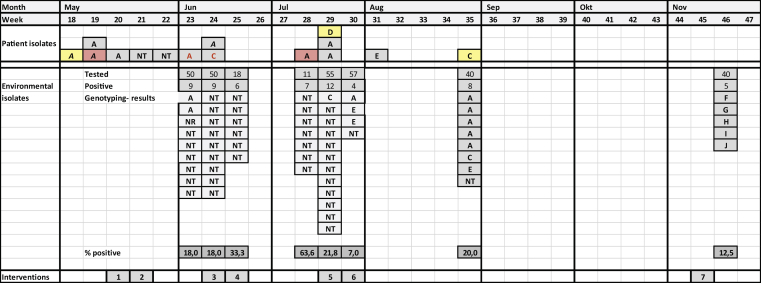
**Epidemic curve ESBL-positive *E.cloacae* outbreak**. Chronologic schematic overview of the epidemic. **Patient isolates**. Colour or rectangle: red; blood culture, yellow; urine, grey: rectal swab. Colour of character: black; haematology patient, red; oncology patient. **Environmental isolates:** 321 samples from various water points were tested; 58 (18%) were positive for ESBL-positive *E.cloacae* with the epidemic phenotype. **Genotyping results (patient and environmental isolates)**: Genotyping results were obtained by AFLP and/or wgMLST. A, C–E: genotype of ESBL-positive *Enterobacter cloacae* strain; italicized = genotyped by wgMLST. NT = not typed. NR = not related. **Interventions**: (1) contact-isolation in separate room for positive patients and all contact patients, (2) start of point prevalence screening of all patients on ward by rectal swab, (3) disinfection of sinks and showers with Na_2_CO_3_ on a weekly basis, (4) replacement of all syphons of sinks on the ward (replacement of shower drains not possible), (5) disinfection of shower drains with chlorine and Na_2_CO_3_ on a daily basis plus closure of culture positive shower drains in the corridor, (6) reopening of showers corridor, (7) termination of weekly point prevalence screening.

The *E. cloacae* outbreak involved 15 patients (13 haematology and two oncology), whom were infected or colonized with a phenotypically identical strain (Figure 1), which proved to consist of several genotypes, found in both patients and the environment. Eventually, a bundle of several interventions, including replacement of siphons and intensive disinfection schemes resulted in the termination of this outbreak. The *P. putida* outbreak occurred one year later and involved nine haematology patients (asymptomatic rectal colonization only). This outbreak was also multi-clonal and involved patients and waterpoints, but this ended without interventions.

## Methods and materials

### Description of water points on the haematology-oncology department

**Figure 2:**
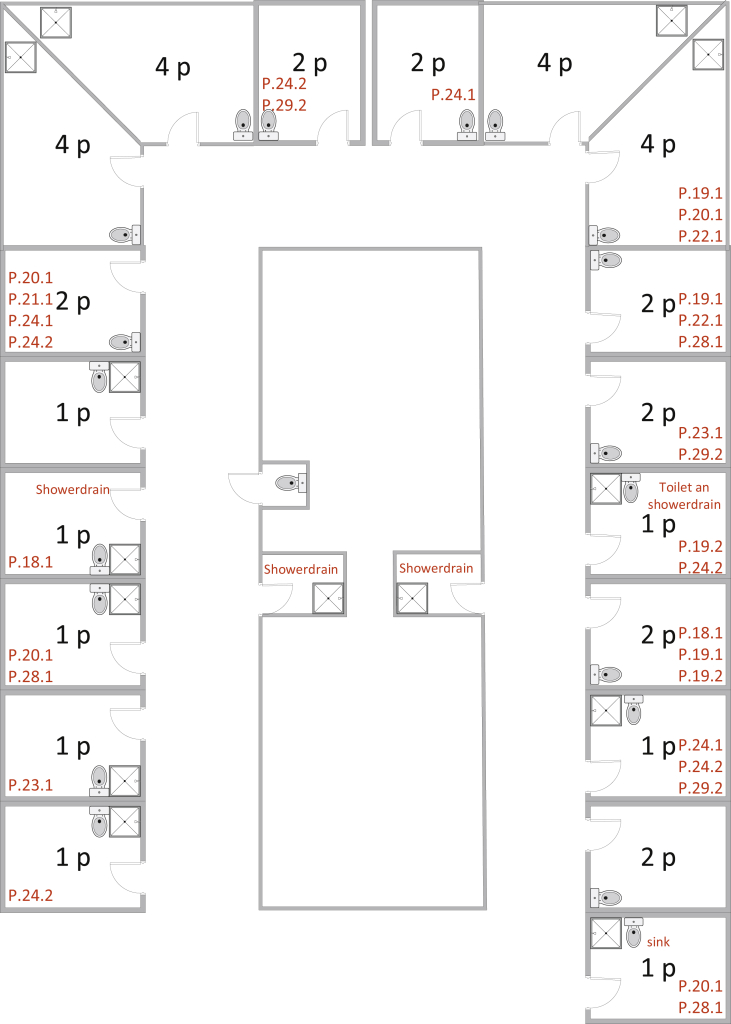
**Overview environmental sampling E.cloacae**. Situation in the haematology department in week 25. In this figure is indicated where patients and environmental samples tested positive for ESBL-positive *E.cloacae*. **Inner rectangles**: 1p/2p/4p = 1, 2, 4 persons patient room. Orange: environmental samples were positive for other Gram-negative bacteria. Red: environmental sample(s) positive for ESBL-positive *E. cloacae* and other Gram-negative bacteria. In the **outer rectangles** is indicated which patients had been admitted to the room before environmental sampling. **Two shower drains were both positive** at the corridor which were used by multiple patients were all positive for ESBL-positive *E. cloacae*.

All single and four-person rooms contain a separate sanitary room containing a sink, shower and toilet. The two-person rooms have a sanitary room with a sink and toilet. For these rooms; two extra shower rooms are created on the corridor of the ward (Figure 2). The sinks are situated directly below the outlet with a siphon. The shower drains are situated in such a way that patients do not stand on it. Before and during the outbreaks, all water points functioned properly.

### Sampling of patients and environment

#### Environmental samples

During the two outbreak periods environmental sampling was performed on the ward, using Copan Transswab (108C USE)®. The interior of sink drains and shower drains were sampled by inserting the swab and rotating at a depth of about 6 cm. Toilets were sampled by swabbing under the edge. Furthermore, faucets and refrigerators inpatient rooms were swabbed.

#### Patient samples

Haematology patients were routinely screened twice a week for colonisation of throat and faeces with Gram-negative bacteria, as a part of their intensive chemotherapy treatment. The oral combination of ciprofloxacin and fluconazole was used prophylactically for SDD, with the aim of preventing systemic Gram-negative or fungal infection during the neutropenic phase of their treatment. If bacteria resistant to ciprofloxacin were cultured from patients, oral colistin was added to bacterial SDD.

During the *E. cloacae* outbreak, present oncology patients were additionally screened weekly by rectal swab for *E. cloacae* carrier status. As no clinical infections occurred during the *P. putida* outbreak, oncology patients were not sampled.

### Microbiological methods

#### Phenotypic investigation

Patient screening swabs were cultured overnight on blood agar and CLED agar at 35°C. As soon as outbreaks were identified, environmental samples and rectal swabs from oncology patients were eluted in Trypton Soya Broth (TSB) and cultured overnight at 35°C. The next day, the TSB was plated and cultured overnight on selective CHROMID® ESBL agar (bioMérieux, Marcy-l’Étoile, France) at 35°C. Bacterial strains were identified and investigated for antibiotic resistance using Vitek-2 (bioMérieux, Marcy-l’Etoile, France). ESBL-production from *E. cloacae* strains was confirmed by the double disk method with cefotaxime, ceftazidime and cefepime with and without clavulanic acid.

#### Genotyping of bacterial strains

*Amplified Fragment Length Polymorphism (AFLP).* Cultured isolates were typed by AFLP analysis as described elsewhere [10]. In brief, isolates were resuspended in 10 mM Tris-EDTA buffer pH 8.0. Restriction was performed with *MseI* and *EcoRI* restriction enzymes (New England Biolabs, Massachusetts, USA), adaptors were ligated and fragments were amplified by PCR with primers specific for the ligated adapters. Following restriction and amplification, DNA fragments were separated on an ABI Prism 3500XL Genetic Analyser (Applied Biosystems, Warrington, UK), after which data were analysed using the Pearson correlation coefficient and were clustered by unweighted pair-group matrix analysis (UPGMA) using BioNumerics software v. 7.6 (Applied Maths, St-Martens-Latem, Belgium). Isolates clustering above the threshold were assigned the same AFLP type number.

*Whole Genome Multi Locus Sequence Typing (wgMLST).* The first cluster of ESL E.cloacae isolates was also typed by whole genome sequencing (WGS). This was performed as described elsewhere [11]. Isolates clustering within an allelic difference of 20 or less were assigned to the same cluster. Sequencing data were deposited at European Nucleotide Archive (ENA) under BioProject PRJEB46126.

### Environmental cleaning and disinfection on the ward

The sanitary facilities of the ward were cleaned daily with moist microfiber cloth and toilets with sanitary cleaner containing alkylalcoholethoxylate, aliphatic alcohol and 4-terr-butylcyclohexylacetate (Taski Sani 100, Diversey, Utrecht, The Netherlands). Floor drains of showers were disinfected with chlorine on a weekly basis for a duration of at least 5 minutes, and toilets were cleaned with fluid containing calcium carbonate (CIF, Unilever, Rotterdam, The Netherlands).

### Experimental study

We investigated whether toilets might have been a driving force behind transmission on the ward. During flushing, droplets have the opportunity to spread to the environment, because the patient toilets in our hospital do not have a cover lid for practical reasons. The experiments were performed in a toilet room of an unused patient department (all toilets in the hospital are identical). *Experiment 1*: 90 mL Visirub® fluorescent concentrate (Hartmann, Nijmegen, The Netherlands) was mixed with the water present in the flushing cistern (estimated volume 9 L). Photographs were taken before and 5 minutes after flushing the toilet, with illumination of the toilet room using UV-light. *Experiment 2*: 24,25 mL Visirub® concentrate was mixed with 24,25 mL demineralized water and 1.5mL gel-former containing polyacrylamide, C13-14 isoparaffin and laureth-7 (Jojoli, Barendrecht, The Netherlands). The resulting fluorescent mixture was applied underneath the toilet edge. Again, photographs were taken in a similar manner.

## Results

### ESBL-positive *E. cloacae*

#### Description of the outbreak [Figure 1]

In May 2019, a cluster of three patients with multi-resistant ESBL-positive *E. cloacae* was detected on the haematology-oncology department of our hospital. The strains were resistant to the empiric antibiotic regimen on the ward (piperacillin-tazobactam) and had an identical antibiogram (ESBL+, ciprofloxacin R, trimethoprim-sulfamethoxazole R). One neutropenic patient had a positive blood culture with this strain, but recovered after switching the antibiotic regime to meropenem. Two of these patients were roommates and all three patients shared the same shower on the ward.

Six new cases occurred in the subsequent five weeks, despite ongoing enforcement of hand hygiene and contact isolation/cohorting of positive patients in a separate room.

By the end of June, disinfection of sinks and shower drains was intensified by replacing all daily and weekly cleaning products with chlorine at 250 ppm. Patients were instructed not to leave their personal belongings on the rim of the sink bowl, to prevent transmission from splashing water from the drain. In addition, all siphons of sinks on the ward were renewed. As environmental samples remained positive for *E. cloacae*, a weekly treatment with biofilm degrading liquid caustic soda (Senzora BV, Deventer, The Netherlands) for five minutes was added for every sink drain and toilet. After this intervention the incidence of newly colonized patients declined gradually over time.

In the peak of the outbreak, a change in the antibiotic regimen of SDD for neutropenic haematology patients, from ciprofloxacin to colistin, was considered an optional alternative intervention. The epidemic strain was resistant to ciprofloxacin, and more than half of the patients on the ward were treated with SDD, so this might have contributed to the rapid dissemination. Eventually, with the decline of new patient cases, this intervention was not effectuated.

A final round of environmental specimens (taken at the end of August) revealed eight positive environmental cultures from which five isolates belonged to genotype A. In retrospect, this could be explained by the suboptimal adherence to the intensified instructions for disinfection by the cleaning personnel. From week 35 on, genotype A *E. cloacae* was not identified in weekly screenings of patients, and environmental samples in November revealed only other genotypes.

#### Environmental samples and patient samples [Figure 2]

In June and July 2019, 47/241 (19.5%) of environmental specimens cultured *E. cloacae* with an identical antibiogram as the initial epidemic strain, among which three were typed as genotype A (three belonged to other genotypes and 41 were not typed). Positive patients were identified in the majority of patient rooms.

#### Genotyping [Figure 3]

All patient strains during the initial outbreak period in May (n= 4) appeared to be identical by AFLP and wgMLST (AFLP-type A). Additional isolates were therefore typed by AFLP only. In subsequent AFLP-analysis there appeared to be the simultaneous presence of multiple genotypes on the ward. In the period May–June, six of seven strains were identical (genotype A). In the period July–August three out of six strains were genotype A.

**Figure 3:**
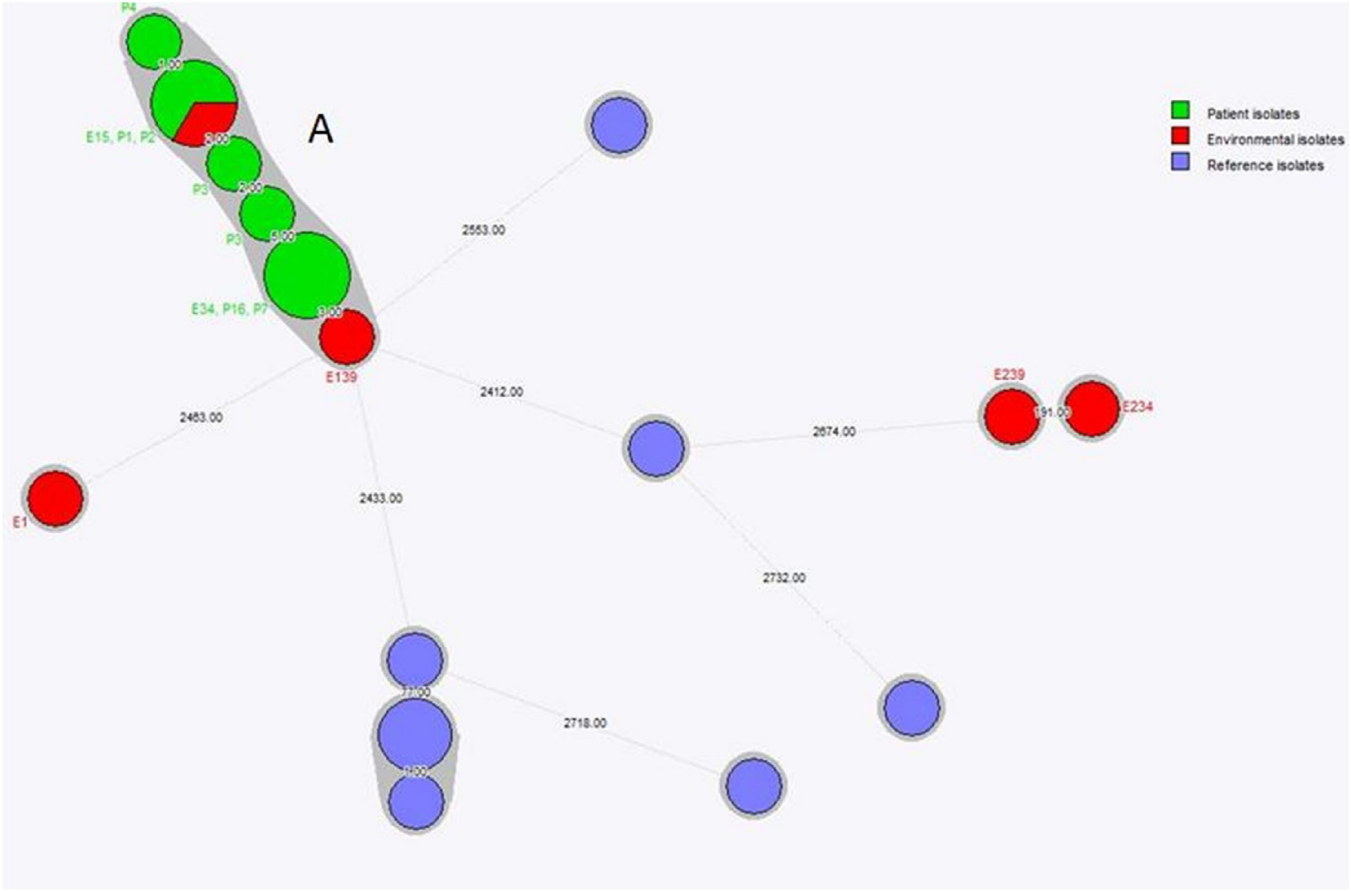
**Whole Genome Sequencing results ESBL-*E.cloacae***. Minimum spanning tree of wgMLST of ESBL-positive *E. cloacae*. Each circle represents one or multiple isolates, depending on the circle size and lines between circles indicate the allelic distance between the indicated isolates. Isolates are coloured by origin: patient isolates (green), environmental isolates (red) and reference non-related isolates (blue). Isolates from patients and the environment that cluster together within the cut-off value are depicted with a grey line zone and indicated as cluster A.

Typing of 18 environmental isolates demonstrated 3/6 genotype A in the period May–July, 7/8 genotype A in August and 0/4 genotype A in November.

### Multi-drug-resistant *Pseudomonas putida*

#### Description of the outbreak [Figure 4]

In August 2020, a cluster of four patients with MDR *P. putida* was noticed in surveillance rectal cultures of haematology patients. The strains were resistant to the empiric antibiotic regimen on the ward (piperacillin-tazobactam) and had an identical antibiogram (ceftazidime I or R, meropenem R, aminoglycosides R, ciprofloxacin R, colistin S).

**Figure 4:**
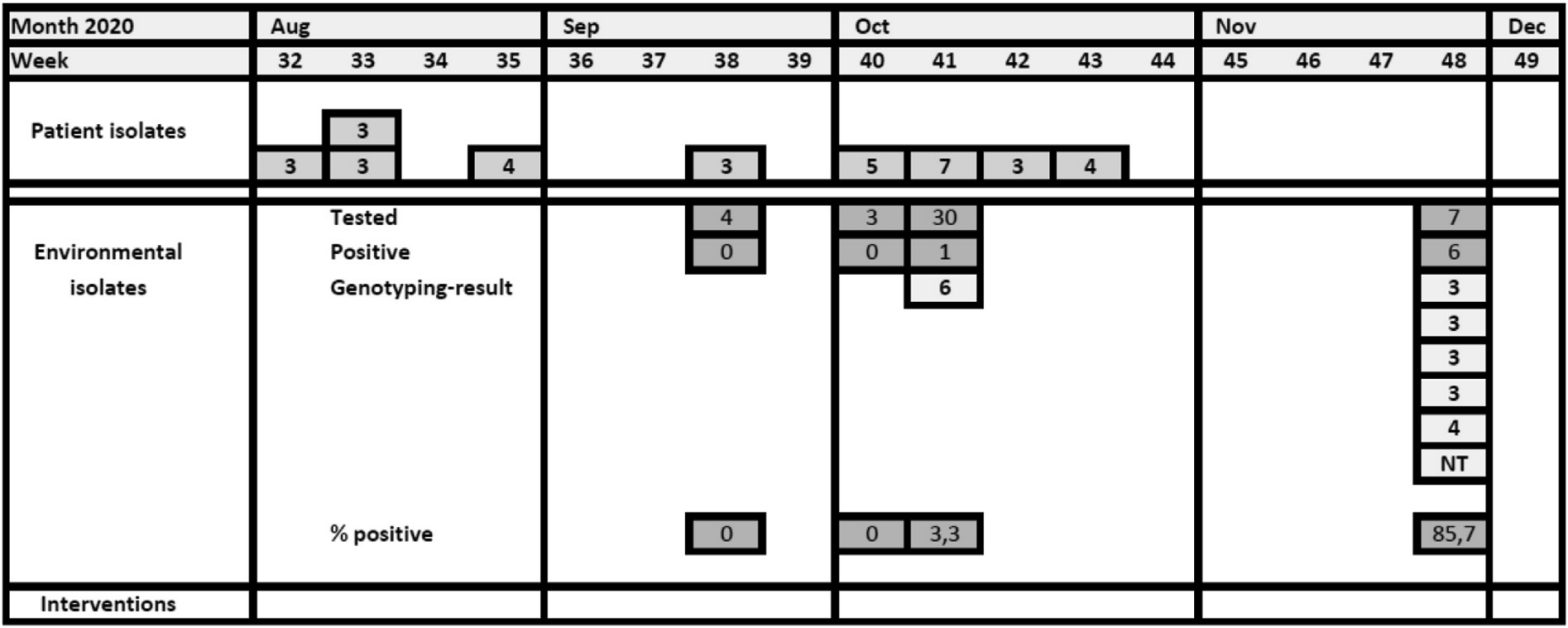
**Epidemic curve multi-resistant *Pseudomonas putida* outbreak**. Chronologic schematic overview of the epidemic. **Patient isolates**. Only positive cultures from rectal swabs for Selective Digestive Decontamination were involved. **Environmental cultures: *week 38***; shower and sink in patient room frequently occupied by positive patients, ***week 40***; ice cube machine; ***week 41***; various water tap points (water from toilets, siphons of basins, buckets used by cleaning personnel), ***week 48***; ridges of 7 toilets from patient rooms of positive patients (note: these cultures were negative for ESBL+ *E.cloacae*). **Genotyping results (patient and environmental isolates)**: 3–7: genotype of multi-resistant *Pseudomonas putida* strain. NT = not typed. **Interventions**: no interventions were carried out.

New cases (n=5) occurred in the subsequent two months, despite enforcement of hand hygiene and contact isolation/cohorting in a separate room of positive patients.

In contrast to the *E. cloacae* outbreak, no clinical cultures became positive with MDR *P. putida*, and the incriminated strains disappeared from rectal cultures of individual positive patients as soon as SDD was changed from ciprofloxacin to colistin. The incidence of colonization reduced spontaneously, so therefore interventions were not necessary. Suspected environmental sources, such as the shower and sink of a patient room that was shared by several positive patients, and the ice cube machine of the ward, were cultured but were negative.

In October, four new patients became positive with a phenotypically identical *P. putida.* Therefore, the 30 toilet edges in all rooms where positive patients had been admitted, were cultured for *P. putida.* Only one toilet edge was positive with a different genotype. It turned out that the four patient strains were different from each other and only one was colonized with the outbreak strain (genotype 3). Since then, no new patients carrying *P. putida* have been identified. Unexpectedly, in the last week of November four out of five genotyped environmental strains from toilets were identified as the epidemic *P. putida (*genotype 3).

#### Environmental samples [Figure 4]

In September–November 2020, 7/44 (16%) of environmental cultures were positive for *P. putida* with an antibiogram identical to the strain found in the cluster in August. From these isolates four belonged to the epidemic genotype 3, two to other genotypes and one was not typed.

#### Genotyping

Three out of four strains of the initial cluster were determined to be identical by AFLP (genotype 3). During the outbreak different AFLP variants were found, from which some were shared between patients and some between patients and environment. Of the new cases in September–October, 2/5 typed isolates belonged to genotype 3 and of the seven positive environmental samples, four were genotype 3.

### Experimental study [Figure 5]

**Figure 5:**
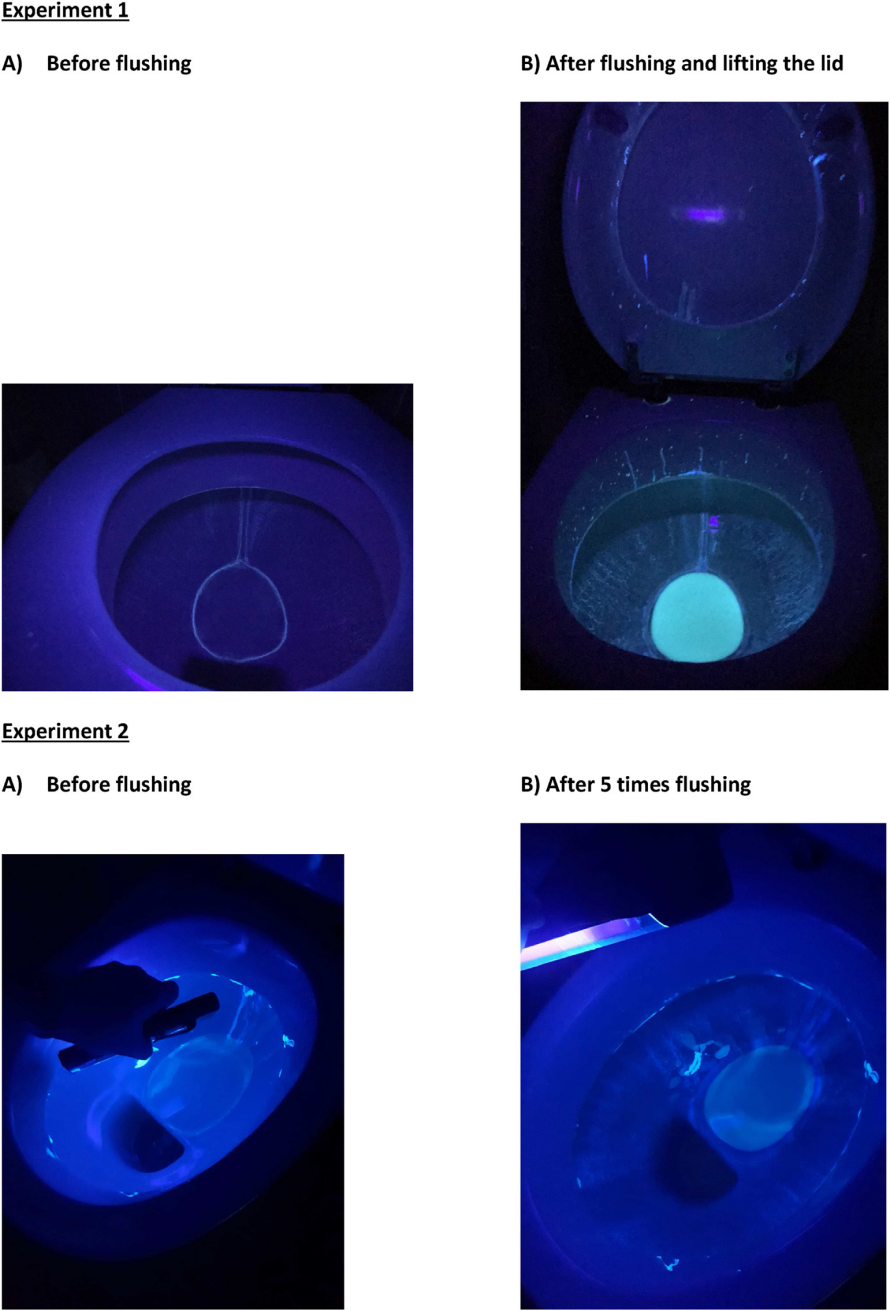
**Experimental study flushing toilet**. Experiments were performed in a toilet room of a unused patient department. *Experiment 1*: 90 mL Visirub® concentrate (Hartmann, Nijmegen, The Netherlands) was mixed with the water present in the flushing cistern (estimated volume 9 L). Photographs were taken before and 5 minutes after flushing the toilet, with illumination of the toilet room using UV-light. UV-positive remainders of droplets were identified inside as well as outside the toilet bowl. *Experiment 2*: 24,25 mL Visirub® concentrate was mixed with 24,25 demineralized water and 1,5 mL gel-former (Jojoli, Barendrecht, The Netherlands). The resulting fluorescent mixture was applied underneath the toilet edge. Again, photographs were taken in a similar manner. Flushing water was able to remove the gel from the toilet.

Experiment 1: fluorescent remnants of flushing water were seen throughout the toilet bowl and some droplets outside were identified (Figure 5A). Experiment 2: flushing water was able to remove some fluorescent gel underneath the toilet edge, which could in theory be further transmitted to the environment by successive flushes (Figure 5B).

## Discussion

In this study we describe the simultaneous colonization of patients and water related devices with ESBL-positive *E. cloacae* or MDR *P. putida* on the haematology-oncology ward of our hospital. A mix of genotypes were isolated in both cases, but a dominant genotype of either *E. cloacae* or *P. putida* was shared by both patients and environment, suggesting that environmental sources played a role in transmission. This hypothesis was supported by experimental results, which proved that by flushing toilets with a fluorescent dye, droplets were clearly disseminated to the environment in toilet rooms. For *E. cloacae*, various interventions resulted in the discontinuation of transmission among patients. For *P. putida*, transmission disappeared without other interventions beside changing SDD from ciprofloxacin to colistin in individual colonized haematology patients.

While the *E. cloacae* transmission was first discovered in clinical cultures of blood and urine, *P. putida* was only found in rectal surveillance cultures*. P. putida* rectal culture positivity was mainly noticed immediately after admission to the ward and disappeared as soon SDD was changed. For both outbreaks, environmental cultures of wet points remained positive for more than a month after the last positive patient was identified. The latter finding implicates that after disinfection of sink or shower drains, biofilm is still present in the deeper compartment of the hospital drainage system. Moreover, recontamination of the water from a biofilm underneath the toilet rim during flushing is a possibility (Figure 5). Bacteria in this biofilm remain an ongoing threat for colonization of patients during their stay in the hospital. As the drainage systems can often not be replaced, the most efficient solution is to “keep the monster inside” by removing the biofilm at the outlets at regular intervals combined with disinfection of these sites. The most efficient and achievable method is still under debate. Recent technical solutions, such as UV disinfection and ozonisation of water seem promising [12]. Innovative design of sinks can reduce the contamination of the near surroundings with bacteria originating from the biofilm [13]. The introduction of self-disinfecting siphons gave excellent results [14,15]. Furthermore, a critical appraisal of the necessity of all water points in the hospital is indispensable; unnecessary ones should be removed and the remaining points should be strictly divided into incoming and outgoing points to prevent cross-contamination [16]. For transmission prevention of highly resistant opportunistic plumbing premise pathogens, design and construction of the water system of newly built hospitals is critical [17]. Also the implementation of waterless patient care is an expensive, but effective method to discontinue hospital transmission [18].

For situations wherein the former solutions are not feasible, the most effective intervention for disinfection and removal of biofilm is still under debate. Recently, Ledwoch *et al.* published a novel in vitro biofilm model to investigate this topic. In a study comparing four methods for disinfection, peracetic acid 4000 ppm was superior to alternatives, resulting in a >4 log reduction in viability in all drain sections [19]. Another weak point in manual disinfection is the correct implementation by the environmental services workers. In the *E. cloacae* outbreak, we discovered that the intensified disinfection strategy was not strictly followed by all members of the team, which contributed to the delayed termination of the outbreak. In this context, not only the type of disinfectant but also the contact time with the various parts of the drains is crucial. Education and feedback by regular audit are essential for high standard cleaning and disinfection in the hospital [20]. Furthermore, we concluded that biofilms do not only occur in the drainage system but also underneath the rim of the toilet seats. Technical solutions, such as rimless toilets, are helpful in prevention of biofilms that are difficult to remove.

High antibiotic use is a well-known risk factor for the selection of resistant bacteria. The majority of patients on the ward were haematology patients receiving SDD, as part of their treatment, for prevention of bacteraemia and mortality. In case of resistance in Gram-negative rods, ciprofloxacin is changed to a combination of trimethoprim-sulfamethoxazole and colistin. As a substantial part of the patient population was treated with an antibiotic for which the *E. cloacae* strains were resistant, this was considered as a possible driving force behind the ongoing transmission on the ward, because the colonization resistance in these patients was lowered. Switching to another antibiotic class could be an effective intervention. Frakking *et al.* used the discontinuation of ciprofloxacin prophylaxis in haematology patients in a bundle of interventions which ended a large and persistent outbreak caused by vancomycin-resistant *Enterococcus faecium*, which is less pathogenic than *Enterobacter* spp [21]. Verlinden *et al.* found no significant increase in serious infectious complications with the discontinuation of prophylaxis and a decrease in fluoroquinolone resistant and ESBL-producing Gram-negative bacteria [22]. Furthermore, the effectivity of ciprofloxacin prophylaxis in preventing mortality is questioned by several authors, and it might even cause selection for multi-drug resistant bacteraemia [23,24].

One limitation of our study is worth noting. We did not prove a causal relation as patients and water points were colonized at the same time period. The index-patient with the *E. cloacae* bacteraemia had been infected on the ward with the same strain one year earlier (data not shown), implying that the environment on the ward could have been contaminated for at least a year. This hypothesis is supported by the fact that at the end of our study in November, a part of the investigated environment was still positive for *E. cloacae*, despite the intensified cleaning and disinfection regime. Recently, Volling *et al.* described a tool for assessing the quality of evidence for transmission from water point to patient. In none of the 52 studies that were evaluated with this tool causality was conclusively demonstrated [25].

## Conclusions

In conclusion, the patient environment is increasingly considered as a major source of transmission of nosocomial bacteria [26]. The drainage system of the hospital is an important reservoir of multi-resistant bacteria, threatening admitted patients. In existing hospitals, biofilms in the drainage systems can often not be removed. Therefore, it is important that in (re)building plans for hospitals a plan for prevention of nosocomial transmission from environment to patients is incorporated. For existing, maybe older, hospital buildings, a water safety plan for haematology-oncology departments should be instituted [27]. Furthermore, where technical solutions are not available, regular training of cleaning personnel is of utmost importance.

## Credit statement of authorship

**WC Van der Zwet:** Conceptualization, methodology, formal analysis, writing original draft, writing review and editing. **IEJ Nijsen:** Methodology, formal analysis, visualization, data curation, writing review and editing. **C Jamin:** Investigation, visualization, writing review and editing. **LB Van Alphen:** Investigation, visualization, writing review and editing. **CJH Von Wintersdorff:** Investigation, visualization, writing review and editing. **AMP Demandt:** Writing review and editing. **PHM Savelkoul:** Writing review and editing, supervision.

## Conflict of interest statement

The authors have no conflicts of interest to declare.

## Funding

No funding was received for this work.

## Ethics and patient consent

Informed consent was not gained from patients involved in this outbreak. All patients were treated according to clinical judgement and infection control practices in order to treat them and control the outbreak according to local guidelines. Patients did not undergo randomisation or intervention for the purpose of this report. Data has been analysed and presented anonymously.
